# Developing a framework of quality indicators for healthcare business cases: a qualitative document analysis consolidating insight from expert guidance and current practice

**DOI:** 10.1186/s12913-019-4269-9

**Published:** 2019-06-28

**Authors:** Myles-Jay Linton, Joanna Coast, Iestyn Williams, Joanna Copping, Amanda Owen-Smith

**Affiliations:** 1 Health Economics at Bristol, Population Health Sciences, Bristol Medical School, University of Bristol, Bristol, UK; 2 The National Institute for Health Research Collaboration for Leadership in Applied Health Research and Care West (NIHR CLAHRC West) at University Hospitals Bristol NHS Foundation Trust, Bristol, UK; 3 Health Services Management Centre, University of Birmingham, Birmingham, UK; 4 Bristol City Council, Bristol, UK

**Keywords:** Business case, Health providers, Quality, Qualitative, Document analysis, National Health Service, UK

## Abstract

**Background:**

Business cases are used to provide a structured justification in favour of investing in new projects, services or interventions. Despite the use of business cases in determining how limited resources will be allocated within England’s National Health Service (NHS), guidance concerning how to develop and evaluate business cases in the context of healthcare is inconstant and of varying relevance. This study aimed to develop a new framework of quality indicators for healthcare-related business cases by analysing the content of expert guidance documents and a sample of NHS business cases.

**Methods:**

Qualitative document analysis was conducted on guidance documents (*n* = 7) and existing NHS business case documents (*n* = 18). Documents were purposefully sampled using criteria to ensure the framework reflected a diverse spread of expert opinion, and a varied sample of example business cases from current practice. Data were analysed using thematic and content analysis, and are presented in a visualised framework.

**Results:**

Seven themes were identified within the qualitative document analysis (purpose, strategic priorities, options, benefits, costs, risks and evaluation). These themes were described and presented with a framework of quality indicators for healthcare-related business cases.

**Conclusion:**

To ou`r knowledge, this is the first framework of business case quality indicators designed specifically for use in a healthcare context. The framework presented in this study has implications for how business cases are developed and evaluated by decision makers. In the future it would be beneficial to investigate how the framework could be used in practice as a tool for critical appraisal.

## Background

Faced with the challenge of increasing healthcare demands on increasingly limited resources, the development and evaluation of business cases provides an opportunity to examine and decide between alternative funding options. Decision makers in many health systems are required to make decisions about how constrained resources will be used optimally [1], yet decisions related to new programmes will vary in the extent to which they are driven by evidence [2–4]. Business cases are routinely used by NHS organisations when considering whether to invest in new services or programmes. The quality of these decisions could be influenced by the quality of the insight that informs them, and numerous procedures, including the use of business cases, contribute to this process. Despite this potential to impact changes in policy, there is an absence of descriptive, collated and informative guidance on the development of healthcare-related business cases.

Business cases are constructed to: outline the rationale and justification for a change [5], secure support and resources from leadership [6], and provide understanding about how a change in practice will yield an economic return on investment [7, 8]. Although business cases continue to be used to support decision making in healthcare settings internationally, questions have been raised regarding the methodological rigor and transparency of these procedures [9]. A ‘good’ business case will provide the detail and clarity necessary for decision makers to make evidence-based decisions, while a ‘poor’ business case may lack persuasion or, in more serious cases, misinform decision-makers about the relative strengths and weaknesses of available options. Subsequently, there is a need to think critically about the use of business cases, what should be included in their ‘content’, and how their quality should be determined.

For clinicians and service providers, business cases provide a potential evidence-based method for instigating changes in practice. Published examples in the literature are diverse and include proposals for embedding the patient perspective within the delivery of healthcare [10], raising standards in infectious disease control [11], part-time employment contracts for nurses [12], art-therapies to manage symptoms of mental health conditions [13], and new technologies for surgical procedures [14]. This diversity in examples illustrates how business cases can be flexibly applied across numerous clinical contexts.

In England, financial strains within the NHS mean that decision makers are required to make funding decisions against a backdrop of increasing pressure to reduce costs and promote efficiency [15]. In the NHS, these concerns are arguably most critical within the Clinical Commissioning Groups (CCGs) responsible for planning and purchasing health services for local populations across England. Decisions concerning these provisions may be influenced by business cases, therefore there is a need for adequate guidance to support their appropriate utilisation.

Generic advice on how to compile business cases exists [12]. It is unclear, however, how well used or useful this guidance has been in healthcare settings. Online resources have been compiled with healthcare in mind [16], and in some cases guidance for writing business cases in relation to specific clinical areas has been published [17]. There is currently no consensus concerning which guidance should be followed; further, most of the available resources do not appear to have undergone any formal process of peer review. Where health-focused resources are available, they have frequently been compiled with specialist clinical areas in mind (such as Parkinson’s or Heart Disease) and may not be more broadly applicable. Given these challenges and the potential for business cases to influence healthcare policy and practice, there is a need to explore the content of available resources, and to analyse the extent to which there are consistent quality indicators that emerge across guidelines, to provide stronger guidance to those putting together such cases.

This study uses qualitative document analysis to develop a framework of ‘quality indicators’, drawing on insights from both available expert guidance documents and a diverse set of existing business cases developed in the NHS.

## Methods

### Design

This study took a qualitative document analysis approach [18], in response to the nature of the data (guideline documents and existing business cases) and the availability of rich, appropriate and previously uncollated written resources. The study team included both academic and professional expertise in applied healthcare research, economics, health services management, qualitative methods, and public health. Where applicable, we adhered to the Standards for Reporting Qualitative Research (SRQR) guidelines to report the findings of this study [19].

### Data collection

We collected guidance documents (GDs), and business case documents (BCs) across CCGs in one geographical region. GDs refer to existing articles and online resources that provide expert advice and support for those either compiling business cases or appraising the quality of existing business cases in the public sector. We collected these documents to capture the ‘best practice’ recommendations made by subject matter experts on the topic. For comparative purposes, we also collected BCs to develop a better understanding of the characteristics of documents used in practice. We supplemented these BCs with documents associated with meetings where BC decision-making was undertaken. Documents were collected on an on-going basis between September 2016 and May 2018. A set of terms were used when searching online for guidance documents (business case, guide, guidance, guideline, checklist, advice, instructions, healthcare and health). This list was informed by terms contained within a research collaboration agreement document developed by a team including expertise in public health, academic research, commissioning, and primary care.

### Document sampling

A set of complementary sampling strategies were employed in response to the evolving nature of the project and structural developments underway in NHS CCGs. Documents were purposefully sampled, with the aim of undertaking a rich and in-depth interpretation, rather than as a systematic attempt to sample documents representative of all guidelines and all business cases.

Guidance documents were selected using criterion sampling. The criteria sought were: (1) documents that were either peer-reviewed publications or online resources, and (2) documents that explicitly listed instructive points for those either developing or appraising business cases. This work was undertaken during a period of ongoing organisational changes, including the introduction of NHS Sustainability and Transformation Partnerships (STPs) across England, therefore it was important to maintain ongoing dialog with identified ‘gatekeepers’ within the CCG to gain access to business cases. Once access to business cases was achieved, individual documents were sampled based on heterogeneity in three characteristics*:* the year the business case was produced, clinical programme area, and funding success.

### Document collection

**Fig. 1:**
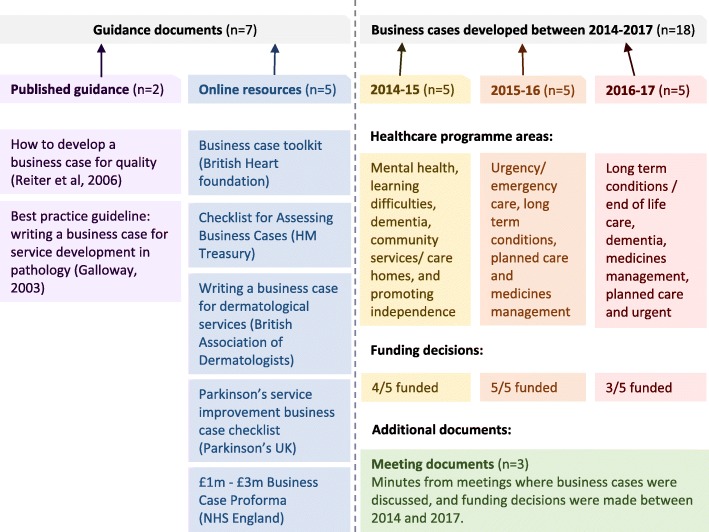
Documents collected for qualitative document analysis

In total 25 documents were collected (presented in Fig. 1). The guidance documents included two peer-reviewed publications [17, 20] and five online resources [16, 21–24]. These online resources were either static webpages or downloadable Microsoft Word/PDF documents. In addition, 15 NHS business cases were sampled from three separate financial reporting periods (2014–15 = 5, 2015–16 = 5 and 2016–17 = 5). All relevant sets of formal minutes detailing decision-making processes and the final funding decisions made at CCG board meetings regarding business cases seeking investment were also examined, to provide further contextual data and enrich our understanding of the topic. Ten of the fifteen business cases were subsequently successfully funded and were on average 14 pages long (range: 3–29 pages). The business cases were diverse in terms of clinical areas (Mental health, Community services, Promoting independence, Long-term conditions, Urgent/emergency care, Planned care, Medicines management, and Dementia), and the type of development they proposed (Investments, Quality improvement, ‘Spend to save’, and Prevention).

### Data analysis

The document analysis approach was undertaken in line with methodological guidance, and combines elements of content analysis and thematic analysis [18]. Content analysis in the context of document analysis refers to the process of identifying and collating meaningful sections of the document text, such as the guidance checklists contained within published papers. Thematic analysis was used to examine how patterns within and between the documents emerge as key themes representative of what ‘good quality’ means within the context of healthcare business cases. Document analyses combine both analytical approaches, to capitalise on the rich breadth of content contained within documents [18], whilst also using a structured approach to handling and organising the data around key topics [25].

The documents were read concurrently as data collection progressed, to develop familiarity with the data. The checklists and recommendations within guidance documents were entered into a Microsoft Excel spreadsheet to enable a structured examination of the data. Business cases were examined in their original format (Microsoft Word or PDF). Throughout the analysis, a log containing notes and emergent ideas was frequently updated. Codes were highlighted throughout the documents to indicate pertinent points. The codes were examined further, and emergent patterns were labelled as potential ‘themes’ for further discussion with the wider study team. Data saturation was achieved when the addition of new documents to the analysis ceased to contribute to the emergence of new themes [26]. The themes and the patterns that supported their emergence were inspected by the wider study team, and any disagreements were resolved through discussions. The findings were reported descriptively and summarised within a visualised framework.

## Results

**Fig. 2:**
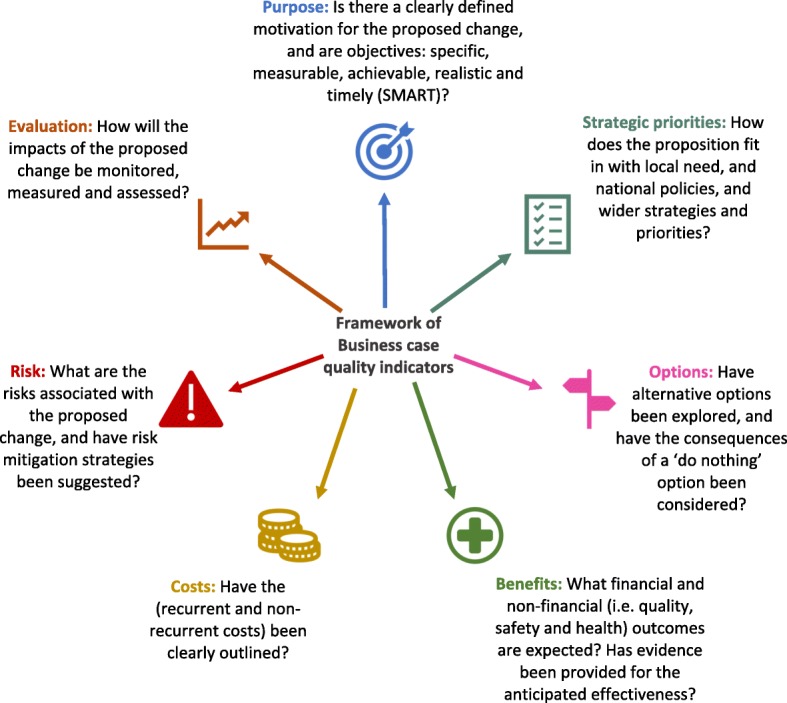
Framework of quality indicators for healthcare business cases

Seven themes were identified: Purpose, Strategic priorities, Options, Risks, Costs, Benefits and Evaluation. These themes are described below, and graphically represented in Fig. 2.

### Purpose

The need for business cases to contain a clearly articulated goal was frequently referred to across most of the guidance documents (5/7). Some guidelines suggested that business cases simply needed to include defined aims and objectives, while others claimed a more structured approach should be taken and any included objectives should be: specific, measurable, achievable, realistic and timely (SMART).

*Ensure your objectives are Specific, Measurable, Achievable, Realistic and Timely (SMART)… Provide a vision of the end position* i.e. *the future you seek to achieve*. (*GD5*).

Despite this, fewer than half of the NHS business cases we analysed (7/15) included explicitly labelled aims or objectives. The remaining business cases described the purpose of the business case in more generic terms as in the following example.

*This business case is to support the implementation of a CCG commissioned Tier 3 Adult Weight Management Service*. (*BC, 2015*).

Although most business cases stated which ‘specific’ demographic would be targeted (13/15), this information was explicitly provided within the objective by only a small number of the NHS business cases.

*The primary aim of the service will be to reduce the incidence of falls in people aged over 65 years and associated healthcare costs*. (*BC, 2016*).

The objectives identified in the analysed business cases also typically lacked ‘measurability’ and instead included unquantified targets as in the following example.

*Sufficient commissioned diagnostic assessment capacity for people referred by General Practitioners*. (*BC, 2014*).

### Strategic priorities

Nearly all the guidance documents stressed that business cases should be positioned within the context of local and/or national strategic objectives (6/7).

*Set the business case within the framework of national and local priorities (the strategic context) (GD3).*

The NHS business cases we reviewed did reflect strategic priorities; however, they were more likely to reflect national rather than local concerns, which were far less clearly articulated.

*NICE Guidelines recommend that older people who present for medical attention because of a fall, or report recurrent falls, or demonstrate abnormalities of gait and/or balance should be offered a multifactorial falls risk assessment*. (*BC, 2016*).

It was clear that many of the business cases were implicitly addressing local needs, such as reducing waiting times. However, only one business case explicitly linked its proposal to a set of local needs.

*Both the [anonymised] Joint Strategic Needs Assessment (2013) and the [anonymised] Health and Wellbeing Strategy (2013) stress the need for diabetes patients to have a universal standard of good care. (BC, 2015).*

### Options

Many of the guidance documents stated that it was important for business cases to clearly outline a range of options for decision makers to consider (4/7).

*Confirm other options considered to achieve the scheme’s objectives*. (*GD7*).

Additionally, some guidelines stated that that one of the discussed options should be a ‘do nothing’ scenario, which deals with the consequences of not implementing any changes.

*Are results of each option presented clearly including do nothing/minimum option? (GD1).*

In contrast to the expectations outlined within guidelines, it was notable that the NHS business cases we reviewed rarely outlined alternative options. However, some of the business cases (3/15) presented partial or full option-appraisals, and explored the benefits and disadvantages of each option, including one scenario where the implications of doing nothing were explored.

*Option 1/Do nothing - CCG(s) to agree how future demand will be managed which cannot be met by the service. Option 2 - Recruit additional staff on a fixed term basis to assist with the waiting list. Option 3 - Recruit additional staff on a permanent basis. Option 4 - Provide a safe service to bariatric patients* (*BC, 2016*).

### Risks

The importance of understanding risks associated with the proposed investment reoccurred throughout the guidance documents (4/7). Beyond simply identifying risky outcomes, a comprehensive business case is expected to outline how these risks will be managed and what process will be put in place to mitigate their impact on the success of the proposed programme.

*The risks have been clearly outlined and a risk mitigation strategy is in place. (GD5).*

All the NHS business cases (15/15) outlined a set of possible risks associated with undertaking the proposed change in service delivery. In most cases (12/15) plans were also outlined to describe how these risks would be mitigated.

*The risk of implementing [programme name] will be mitigated through introducing the new service as a ‘test and learn’ pilot based initially in one GP cluster area. (BC, 2016).*

‘Monitoring’ implementation was the most frequently stated method of mitigating risk.

*Capacity – there has not been a service like this before in [anonymised region] so it is difficult to predict demand. Mitigation = Monitor referrals to ensure appropriateness & regularly review workload and project outcomes. (BC, 2015).*

In some business cases however, there was a greater emphasis on the need to provide more explicit points surrounding how these risks would be tackled, above and beyond monitoring adverse outcomes.

*The risks of implementing a Tier 3 service will be mitigated through introducing the new service as a Test and Learn pilot. This will allow the CCG to test and evaluate the new Tier 3 model (the demand, costs, outcomes) before a full procurement exercise takes place for a more permanent solution. (BC, 2015).*

### Costs

Almost all the reviewed guidelines documented several ways in which the costs associated with business cases should be evidenced and presented (6/7). Understanding what is already funded, quantifying and itemising costs, and clarifying where funds would come from were flagged as important considerations.

*Consider finances - Be clear on what services your organisation currently pays for, how much any improvements will cost and where the funding will come from. (GD2).*

Within one guideline, costs and potential benefits were considered simultaneously.

*Are all economic costs and benefits clearly calculated for each year covered by the proposal with Net Present Value (NPV) calculated correctly? (GD1).*

All of the analysed business cases (15/15) presented numerical figures for how much investment their initiatives required. Some of these business cases outlined the cost attached to employing a new member of staff, while more detailed cases outlined a more in-depth breakdown of costs as in the following example.

*Training in exercise for back care (£600), Co-ordinator/provider 0.5 WTE for 9 months (£11,820), Venue costs (£3640), Co-ordinator travel costs (£250), and Contingency (*e.g. *sickness cover) (£500), and Total (£16,810). (BC, 2016).*

### Benefits

All the guidance documents described alternative ways of detailing expected benefits, in terms of the positive outcomes resulting from investment. Within these documents, benefits were predominantly discussed in terms of cost-savings or quality improvements.

*Confirm the scheme benefits – including financial (cash releasing and non cash releasing) and non financial (quantifiable and non quantifiable) and how the scheme delivers value for money. (GD7).*

One guideline document also highlighted the need to be realistic about the estimated benefits, in light of known and unknown threats.

*Is optimism bias properly included and aligned with risk? (GD1).*

Within existing business cases, benefits were consistently predicted in terms of financial consequences.

*Savings from such investments elsewhere on the same scale as proposed for [anonymised region] have delivered savings between £83,580 and £585,000. (BC, 2016).*

In other cases, benefits were also descriptively expressed in terms of improvements to the service provided specifically to patients.

*Maximise a patient’s physical and psychological health through lifestyle advice and education on medication, exercise and breathlessness. (BC, 2015).*

### Evaluation

A small number of the guidance documents highlighted that it was necessary for business cases to include plans for identifying positive and negative outcomes if the planned initiative was successfully funded (2/7).

*Does the plan include post implementation evaluation arrangements (including who, when, how, and costs)? (GD1).*

Although evaluation was highlighted in a minority of guidance documents, most of the business cases proposed ways in which their service could be monitored and subsequently evaluated (11/15). These evaluation plans differed in terms of: what was monitored, who would be involved in the evaluation, what type of analysis would be undertaken and the time periods that the evaluation would be conducted within.

*The Tier 3 service will be asked to collect and report on a range of variables on each patient to demonstrate success at 6 and 12 months. (BC, 2016).*

### Framework of quality indicators for healthcare business cases

The seven qualitatively derived quality indicators are presented within the framework in Fig. 2. Although quality indicators have been highlighted and described individually, they should be viewed as distinct yet inter-related concerns. For example, a business case would be enriched if it discussed the associated ‘risks’ when outlining alternative ‘options’. Further, the framework does not describe a sequential order in which the quality indicators should be addressed. In practice, questions surrounding how the impact of a business case’s proposal should be ‘evaluated’ should be considered in advance. Further, the ‘purpose’ of a proposed change in practice should be clear throughout a business case, not simply at the beginning.

## Discussion

### Main findings and interpretation

This document analysis has identified and amalgamated insight from expert guidance and current practice to develop a set of key quality considerations for the development of business cases in healthcare settings (Fig. 2). The seven themes derived from this analysis (purpose, strategic priorities, options, benefits, costs, risks and evaluation) have been organised into a framework to support the development and interpretation of business cases. To our knowledge, this is the first framework of business case quality indicators, designed specifically for use in a healthcare context. The extent to which the NHS business cases we analysed addressed these quality indicators varied. For example, beyond simply stating what the risks of the proposed programme are, greater quality was indicated by also outlining how these risks will be mitigated. Similarly, beyond presenting the proposed service as the only option, the more comprehensive business cases detailed alternative courses of action, including the consequences anticipated if ‘no change’ were to be implemented. Longer business cases were not necessarily any better at providing full coverage of the quality indicators, indicating that length alone does not necessarily guarantee quality. Consequently, it would be beneficial to structure the development of business cases around a defined set of critical quality considerations, rather than writing at length without clear focus on the most useful information for decision-making.

One of the key links between this research and the wider literature on healthcare improvements is the importance of evaluation in the context of implementation. Evaluative evidence should influence changes in practice [27], however this research also highlights that it is important to evaluate the implementation of changes to healthcare practice, to assess the occurrence of expected (and unexpected) outcomes. Evaluation methods should be appropriately tailored to improvement initiatives [28], with plans outlined to describe how any evidence generated will inform future practice. Comprehensive guidance on the use of quantitative and qualitative methods to capture outcomes and lessons learned as innovations are being implemented is available in the literature [29], and could be used in connection with the model proposed in this research.

In this study, the strategic priorities within the NHS business cases that we analysed were more likely to reflect regional strategies such as those outlined within Joint Strategic Needs Assessments [30], rather than international objectives [31]. Intuitively, business case authors may choose to emphasise how their intended innovations match the scope of priorities they anticipate decision-makers responsible for funding will be most focussed on (in this case the objectives of NHS clinical commissioners). Although heterogeneity in the challenges prioritised across health-systems is expected, numerous global public health concerns, such as the successful management of antimicrobial resistance require coordinated and collective action from healthcare providers internationally [32].

### Implications

These findings have implications for how health-related business cases are compiled, evidenced, and evaluated. This analysis suggests that authors developing business cases should prioritise the inclusion of information and evidence concerning the purpose, strategic priorities, options, benefits, costs, risks and evaluation of a service or programme. Although use of this framework in practical settings such as NHS CCGs has currently not been tested, constructing business cases in line with these quality indicators provides a structured way of addressing the concerns highlighted by several sources of best practice expert advice. Through this process of critical reflection, business cases may be strengthened through greater clarity, or undergo comprehensive redevelopment.

### Strengths and limitations

The main strength of the research was its novel integration of both top down (expert guidance) and bottom up (existing business cases) influences. The integration of expert guidance harnesses best-practice knowledge, and the influence of existing business cases ensures that the framework reflects examples from current practice in decision making (external validity). The use of document analysis minimises the opportunity for ‘researcher contamination’ in the production of data as (unlike interviews and focus groups) documents are exact and have stable content [18]. Conventionally, qualitative studies are rarely exclusively dependent on documents as data, however, given this study’s focus on written business cases, document analysis combined with thematic analysis provided an appropriate and flexible methodological foundation for interpretation.

One limit on the generalisability of the study is that the NHS business cases we analysed were developed in one geographical region. Due to the confidential and sensitive nature of business cases, it was not feasible to source documents from numerous CCGs. The use of Clinical Commissioning Group business cases also indicates that further work is needed to explore and incorporate a focus on public health business cases developed externally to the NHS. Additionally, it would be informative to explore the relevance of this model across health systems internationally. Further, we collected business cases retrospectively, therefore we relied on the content of meeting minutes to gain a deeper understanding of the decision-making context. Observing the meetings first-hand would have provided a richer understanding of the decision-making process and should be explored in future research. Finally, although we were able to develop a framework informed by a rich and in-depth qualitative analysis, we did not use systematic review methods to collect the documents providing data, which is a recognisable methodological limitation.

### Future research

The framework of quality indicators presented in this paper has been developed in accordance with available expert guidance and existing business cases, however there remains a need to test the use of the framework in practice. Future study might therefore qualitatively explore the addition of other, currently absent, quality indicators. In this way, researchers may move beyond focusing on how evidence fails to get into practice and towards responding to the complexity of decision-making and exploring the ways to facilitate the mobilisation of knowledge and evidence [33]. Given the recognised importance of critical appraisal tools for assessing quality [34], a worthwhile focus for future study would be examining the feasibility of structuring a critical appraisal checklist for healthcare business cases around the emergent themes from this research. It would also be beneficial to explore the role that contextual factors (such as organisational culture and capacity) might play in dictating the success of funded business cases once plans are implemented into practice.

Although business cases are used to allocate resources in numerous healthcare settings internationally, alternative decision-making strategies exist. Considering the varying quality of the business cases in use, future work is required to explore whether business cases are an appropriate method for allocating healthcare resources. Although business cases provide flexible and ad hoc opportunities for innovation, there may be more efficient and evidence-based methods for introducing service improvements. In future research, it would be interesting to compare and contrast the use of business cases with other strategies such as Programme Budgeting and Marginal Analysis [35] or Multi-Criteria Decision Analysis [36], although these methods tend to be used as part of a comprehensive priority setting exercise when a number of options are considered simultaneously, rather than the more piecemeal decision-making that might be associated with use of business cases. Such research would help to build a more comprehensive and comparative understanding of the strengths and weaknesses of these alternative approaches.

This research highlights multiple, potentially competing priorities to be addressed within the development of healthcare business cases. Further research is required to investigate how decision makers trade off on the importance of these multiple competing priorities, and the rationale that drives this process of prioritisation. For example, how do CCGs understand and balance pressures to innovate with the need to address local population needs and demands (e.g., using evidence within Joint Strategic Needs Assessments), whilst also factoring in resources to evaluate the impact of changes in practice. Discrete choice experiments could be used to examine stated preferences among different stakeholders (healthcare practitioners, decision makers and patients) around the importance of evidence for each of the quality indicators within the framework presented in this paper [37].

## Conclusion

The use of business cases in healthcare facilitates the allocation of limited resources to services and programmes presented in the form of comprehensive evidence-based proposals. This study provides a set of qualitatively derived key quality indicators for clinicians and decision makers tasked with constructing and interpreting business cases. Future work to investigate the feasibility and potential usefulness of developing this framework into a critical appraisal checklist would have further implications for practice and policy. Future research should explicitly investigate the extent to which healthcare decision-making is enhanced through the use of business cases influenced by the guidance presented in this paper, or whether other approaches should be encouraged.

## Data Availability

The data that support the findings of this study are available from (Anonymised) CCG but restrictions apply to the availability of these data, which were used under license for the current study, and so are not publicly available. Data are however available from the authors upon reasonable request and with permission of (Anonymised) CCG.

## Abbreviations

BCs: Business cases
CCGs: Clinical Commissioning Groups
GDs: Guidance documents
NHS: National Health Service
SMART: Specific, Measurable, Achievable, Realistic and Timely
SRQR: Standards for Reporting Qualitative Research
STPs: Sustainability and Transformation Partnerships
